# China’s Pathway towards Solar Energy Utilization: Transition to a Low-Carbon Economy

**DOI:** 10.3390/ijerph17124221

**Published:** 2020-06-13

**Authors:** Muhammad Awais Gulzar, Haroon Asghar, Jinsoo Hwang, Waseem Hassan

**Affiliations:** 1 University of Waikato Joint Institute, Zhejiang University City College, Hangzhou 310015, China; awaisopf@hotmail.com; 2 Waikato Management School, University of Waikato, Hamilton 3240, New Zealand; 3 Department of Agriculture Economics and Management, Zhejiang University, Hangzhou 310058, China; 11622058@zju.edu.cn; 4 Department of Food Service Management, Sejeong University, Seoul 143-747, Korea; 5 NUST Business School, National University of Sciences and Technology, Islamabad 44000, Pakistan; waseem.hassan@nbs.nust.edu.pk

**Keywords:** low-carbon economy, solar energy, environment, emission, development

## Abstract

Rapid economic growth has caused many environmental problems in China, resulting in international pressure on China to fight against climate change and to shift to a more environmentally friendly economy. Therefore, over the past decades, China has been working on transforming its economy to counter the concerns of different environmental hazards caused by the burning fossil fuels and rising oil imports to support the energy sector. This study explores the shift in the Chinese government’s policies towards a low-carbon economy by adopting more environmentally friendly solar energy. A cost–benefit analysis of the solar water heater industry in China indicates that it not only brings economic benefits to society but also environmental benefits to the earth. Furthermore, this paper focuses on the use of solar energy as a kind of renewable energy, as solar energy is plentiful, which is beneficial from both an environmental and economic perspective.

## 1. Introduction 

In developing countries, economic growth is vital. However, environmental conditions have always been ignored. In China, economic growth has caused many environmental problems [1]. A great amount of fossil resources are used in producing goods, so these production processes emit a lot of greenhouse gases, which increase the global warming effect. As more and more people in China own private cars, these cars require a great deal of oil, and the automobile exhausts are high in carbon [2]. In the last two decades, the shift toward a low-carbon economy has played a vital role in adopting green energy economics. Since 1990, there has been a major increase in studies conducted relating to the area of the low-carbon economy [3]. In recent years, the concept of a low-carbon economy is widespread in China. People have begun to balance economic growth and good environmental condition. In order to reduce the emission of high carbon gases, low-carbon energy is preferred, such as renewable energy [4]. Technology innovation has been used to develop a renewable energy industry and to increase the efficiency of energy use. Solar energy is the most common renewable energy, and is available in all parts of China. The solar energy industry is developing rapidly in China, and it plays an important role in achieving a low-carbon economy [5,6]. 

The solar energy heat utilization industry and the solar photovoltaic industry are the two main parts of the solar energy industry. The development of the solar energy heat utilization industry has been significant recently in China [7]. The solar water heater is a popular product in China. Increasing numbers of people can now receive both the economic and environmental benefits of a solar water heater. From a macro and micro view of the solar water heater industry, the benefits of this industry exceed its costs [8]. The cost–benefit analysis shows that this industry can help save a lot of non-renewable energy. Although solar energy can be used without emitting greenhouse gases, and is abundant and low cost, there still exist some shortcomings of this energy. The process of producing a solar water heater will emit carbon gases [9]. Furthermore, solar energy is not very stable, so storage machines should be made to save the energy. Finally, the solar collection machine can cause light pollution. Some advanced technology is still needed to solve these problems of using solar energy and to increase the contribution of the solar energy industry toward reaching a low-carbon economy [10,11,12]. 

This paper will examine the pathway towards a low-carbon economy by solar energy in China. There are mainly two different solar energy technologies, solar photovoltaic (PV) and solar water heaters (SWH), in china. Based on REN21’s 2017 report, renewable energy has contributed 19.3% to humanity’s global energy consumption and 24.5% to their generation of electricity in 2015 and 2016, respectively. This energy consumption can be further divided into 8.9% from traditional biomass, 4.2% as heat energy (modern biomass, geothermal and solar heat), 3.9% from hydroelectricity and the remaining 2.2% from electricity from wind, solar, geothermal and other forms of biomass.

## 2. Literature Review

For the past 200 years, fossil fuel has been used as the main energy driver for the growth and development of economies. The role of fossil fuel in growth and development has been well documented by researchers of long-term history as well as of modern day economic drivers of growth [13,14,15,16]. The main goal of modern energy sources for economic development are to give easy access to affordable, sustainable and modern energy sources [17]. Therefore, the main emphasis is on the creation and adoption of energy sources with low-carbon emissions, so that the natural environment is not harmed by them.

Joseph Schumpeter, in his book *The Theory of Economic Development,* laid the foundation of our understanding of the economy as a dynamic and evolving system centered innovation and new technology. He emphasized sources of energy creation as an integral part of economic development [18]. Many ecological economists and developmental economists have urged the aspect of adopting low-cost and high quality energy inputs as a source of economic growth [19,20,21,22]. They emphasized different energy forms as source of energy through coal, oil and gas, which have a significant contribution to economic output but also have some environmental hazards, which should also be addressed while adopting those sources of energy. To achieve economic growth and development along with the creation of low-carbon sources of energy, economists have given a transition pathway to a sustainable low-carbon economy; their research emphasized alternatives for socio-technical energy systems and the adoption of infrastructures for a low-carbon future for economies [23].

### 2.1. Definition of a Low-Carbon Economy

A low-carbon economy means using fewer natural resources and releasing less pollutants to gain more economic output. The characteristics of a low-carbon economy are low energy consumption, low carbon emissions and low environmental pollution [24]. According to Zhuang [25], the essences of a low-carbon economy are the highly efficient utilization of energy resources and the construction of clean energy resource structures. In his opinion, the main methods of achieving a low-carbon economy are energy resource innovation and regulation innovation. In addition, a low-carbon economy compromises two aspects: using modern technology to achieve energy conservation and improve utilization efficiency and the development of new sources of energy [26]. A low-carbon economy can be regarded as a new economic, technological and social system; based on the thoughts of Yan, Zhang, & Shen [27], this new system has a lower level of energy consumption and greenhouse gas emission in the production process and consumption, whilst keeping the development of the economy and society [28]. 

### 2.2. Definition of Solar Energy

Solar energy is renewable energy, and it is also a kind of no-carbon energy. Everyone can use the energy without paying fees, and there is no need to transport solar energy [29]. Solar energy comes from the radiations of sun, and people can use this energy to produce heat and electricity [30]. 

### 2.3. Relationship between the Solar Energy Industry and a Low-Carbon Economy

The solar energy industry is mainly focused ton two fields: the solar energy heat utilization industry and the solar photovoltaic industry [31]. Solar energy is a kind of clean resource which can reach the requirements of a low-carbon economy, because when using solar energy there are no pollutants being released into the environment [32]. In China, 70% of all energy is sourced from coal, which leads to high carbon emissions [33]. Solar energy can replace fossil resource energy. As the rural population is large, the solar water heater can be widely used in the rural areas in China so that a significant amount of carbon emission can be reduced [34].

## 3. Data Analysis

### 3.1. Solar Energy Allocation in China

There is abundant solar energy in China. In most parts of China, the amount of solar radiation is more than 4 kwh (kilowatt hours) per square meter every day, and in some areas this amount is 9 kwh per square meter per day [35]. The average number of sunshine hours in different cities is variable. According to Table 1, the average daily sun shine hours are greatest in Lhasa, with 6.70 h, and is smallest in Guiyang, with 2.84 h. Solar energy covers all the land of China, so it is easy to collect solar energy in any part of China [5,36].

### 3.2. Cost–Benefit Analysis of the Solar Water Heater

The solar water heater industry is developing rapidly in China. More and more families are choosing a solar water heater because of its low cost and low-carbon emissions [38]. As the low-carbon economy is related to the economy and the environment, a cost–benefit analysis a good method to show the low-carbon characteristics of the solar water heater [39,40]. There are macro and micro analyses of the solar water industry in the following part. At the macroscopic level analysis of solar water heater industry, there are plenty of benefits in the nationwide range. As the data in Table 2 shows, the total environmental benefit of using a solar water heater, per square meter, over the course of its lifetime, is 750.2 CNY. Using a solar water heater can help reduce the emission of SO_2_, NO_2_, smoke dust and CO_2_. The solar water heater industry generated increasing revenue between 2006 and 2010, as shown in Table 3, and this industry has also increased exports during this period. At the same time, the solar water heater industry also provided more and more job opportunities between 2006 and 2010. Table 4 shows that, in 2010, employment in the solar water heater industry in China was 3,300,000 persons. As seen in Table 5, the environmental benefits of solar water heaters showed an increasing trend between 2006 and 2010 in China. The emission of harmful and greenhouse gases has been significantly reduced. In addition, solar water heaters also save a great amount of coal and electricity. All the data show that solar water heaters not only reach the requirement of low carbon emissions, but also have many economic and social benefits for the whole nation [41,42,43].

In the microscopic view of the solar water heater industry, a cost–benefit analysis can be performed in the small family range. Assume that a family of two people buys a water heater. If they choose a solar water heater, the costs of the solar water heater are the purchase price, maintenance fees and water fees. The benefits of a solar water heater are the environmental benefits and economic benefits [45]. 

Before calculation of the total costs, the basic information is as follows. According to information on the Amazon [46], the purchase price of a solar water heater is ¥3500, the useful lifespan is 10 years and the area of the solar energy collector is 1.96 m^2^. According to solar water heater company Huangming, the solar water heater is guaranteed for three years, with 10-yuan maintenance fees, and the next seven years’ maintenance fees are 30 yuan per annum. The water price is 1.85 yuan per ton [47]. Every year, assume that each person takes 260 showers, so the total number of showers taken in this family is 520. Each person will consume 0.2 tons of water when taking a shower. Thus, the annual water fee is ¥192.4 (520 × 0.2 tons × ¥1.85/ton). The discount rate of costs and benefits refers to the deposit rate of the Bank of Hangzhou, which is 0.385% (Deposit, 2012), because the solar water heater can be regarded as a kind of investment. As Table 6 shows, the present total cost of using a solar water heater is ¥5604.67.

Apart from the costs of the solar water heater, the environmental benefits can be calculated on a monetary basis. The environmental benefit of reducing the emission of CO_2_ is the largest. Table 7 shows that the total annual environmental benefit is 264.48 yuan for this solar water heater. The present value of the total environmental benefit is 2589.67 yuan, as shown in Table 8.

However, if this family chooses an electronic water heater, the market price is ¥1756 and the annual maintenance fee is 40 yuan, based on the price of Amazon [48]. The electronic fee is 0.5 yuan per kwh; every time they take a shower, each person will use 0.7 kwh, so the electronic fee every time is 0.35 yuan (Zhang, 2008). The annual electronic fee of this family is 182 yuan (520 × 0.35 yuan/time). Table 9 shows that the present value of the total-use costs of an electronic water heater is 5806.85 yuan. Compared with a solar water heater, an electronic water heater costs an extra annual electronic fee of 182 yuan. The present value of ten years’ electronic fees is 1782.05 yuan. Thus, using a solar water heater can save 1782.05 yuan of electricity. This can also be regarded as a kind of benefit of using a solar water heater. 

This family has a third choice, which is to use a coal gas water heater. The purchase price of a coal gas water heater is ¥2000; each person will use 0.5 m^3^ coal gas every time of shower, and the coal gas price is 0.79 yuan per m^3^ [49]. The annual coal gas fee is therefore 205.40 yuan (520 times × 0.5 m^3^/time × 0.79 yuan/m^3^). The present value of the coal gas fee is 2011.17 yuan, which is shown in Table 10. The present value of the total cost of using a coal gas water heater is the largest. A solar water heater can save the coal gas fee, compared to the coal gas water heater. Therefore, the solar water heater also has the benefit of 2011.17 yuan, which is the value of the coal gas saved.

The solar water heater can gain the benefits that are reducing use of electricity valued of 1782.05 yuan and avoiding using coal gases valued of 2011.17 yuan. These benefits can be considered as a kind of economic benefits of this family which are 3793.22 yuan in total. In the other side, these benefits can also have been regarded as energy conservation which is a contribution to the low-carbon economy. Total present value of the environmental and economic benefits of solar water heater is 6382.89 yuan (3793.22 + 2589.67) which is larger than the present value of total costs 5604.67 yuan. Based on the cost-benefit analysis, solar water heater is a low-carbon product that is helpful in developing low-carbon economy. 

## 4. Findings 

The most salient feature of solar energy is that solar energy is a truly renewable energy source. Solar energy can be harnessed all over the world. Therefore, solar energy is considered to be a solution to energy crises around the world because other sources of energy are being created by using non-renewable resources, which may run out with the passage of time, whereas the source of solar energy is sunlight, which will be available to us for at least 5 billion years, according to scientists [50]. In the majority of the areas of China, solar energy is available in abundance, and people can collect solar energy directly and produce electricity and heat by solar energy.

The second advantage is in the reduction of monthly electricity bills; solar energy is considered a one-time investment. A person has to invest once to buy the equipment, and afterwards there is a very small maintenance cost associated with solar energy, whereas fossil fuel energy has a monthly cost, which can take a major portion of monthly income.

The third advantage is that solar energy is clean for the environment. There is no by product from solar energy, such as waste solids, waste water or waste gas [51]. It is considered to be the most cost efficient and environmentally friendly energy because of its low carbon emissions.

The results also give us the insight that a comprehensive policy framework is needed to sustain energy efficiency improvements in China. The most essential policies are those targeting improvement in the pricing mechanism of the energy sector by the development and upgrading of renewable energy markets and raising awareness regarding the future prospects of renewable energy as a pathway to a low-carbon economy.

### Disadvantages of Solar Energy

Although solar energy is a clean energy and is easy to access, there are still some disadvantages to it. As is known, people use tools and machines to collect solar energy and gain the benefits of this energy. The process of producing solar energy collection machines in China requires a great amount of non-renewable energy, such as metal materials [52]. These metal materials are processed using oil and coal energy, which lead to high carbon emissions. From this point of view, using solar energy is not absolutely no-carbon emission. 

In addition, solar energy is not a very stable source of energy. In different seasons, different times of a day and in different parts of China, the strength of solar energy is not the same [53]. On rainy days, it is hard to collect solar energy because solar energy is heavily dependent upon the input source of sun, and in winter and on rainy days it is very difficult to get the solar panels to work to their maximum; therefore, the efficiency and effectiveness of the solar panel generated energy is compromised. This is why solar panels are usually installed in houses in china as a secondary source of energy, rather than the primary source.

Finally, light pollution is another disadvantage of using solar energy. The collector of a solar water heater will reflect sunlight, leading to light pollution. Light pollution is harmful to people’s eyes and causes cataracts [54]. Light pollution also results in headaches, losing sleep etc. 

The manufacturing of solar panels involves the use of materials that are hazardous to human beings and nature, such as sulfuric acid and phosphine gas, which are very difficult to recycle. According to environmental progress analysis, manufacturing solar panels results in the creation of about 300 times more toxic waste per unit of generated electricity than a nuclear power plant [55]. There is a great risk of hazardous material being transmitted into the environment from the by production as industrial waste from the solar panel industry. Solar panels contains toxic metals such as cadmium, which are known to be carcinogenic, as well as lead, which can damage the nervous system. Both of these metals are leached out of waste dumps into drinking water, causing fetal and incurable diseases to people drinking the affected water [56].

## 5. Conclusions

### Policy Implementation

The low emissions and low cost of solar energy contributes to the process of reaching a low-carbon economy. The cost–benefit analysis of the solar water heater industry in China indicates that solar water heaters not only bring economic benefits to society, but also environmental benefits to the earth. As a kind of renewable energy, solar energy is plentiful. Using solar energy does not create carbon emissions. However, the production of solar energy collection machines is not low-carbon, and some non-renewable energy is still used, with emissions of carbon gases. The fluctuation of solar energy is also a shortcoming, which requires energy storage techniques to save the solar energy. All in all, the solar energy industry still has positive effects on the development of a low-carbon economy. From a more practical perspective, policy makers should not only be concerned with energy generation and renewable energy; they should also focus on the environmental aspects with regards to renewable energy as a source of low-carbon energy that will have a much greater impact on the cleaner environment.

## Figures and Tables

**Table 1:** Average sunshine hours per day in provincial capital cities in China.

City	Latitude (North)	Average Sunshine Hours per Day	City	Latitude (North)	Average Sunshine Hour per Day
Harbin	45.68	4.4 h	Hefei	31.85	3.69 h
Chuangchun	43.90	4.80 h	Shanghai	31.17	3.80 h
Urumchi	43.78	4.60 h	Chengdu	30.67	2.87 h
Shenyang	41.77	4.60 h	Wuhan	30.63	3.80 h
Hohhot	40.78	5.60 h	Hangzhou	30.23	3.42 h
Beijing	39.80	5.00 h	Lhasa	29.70	6.70 h
Tianjin	39.10	4.65 h	Nanchang	28.67	3.81 h
Yinchuang	38.48	5.50 h	Changsha	28.20	3.22 h
Taiyuan	37.78	4.80 h	Guiyang	26.58	2.84 h
Xining	36.75	5.50 h	Fuzhou	26.08	3.46 h
Jinan	36.68	4.44 h	Kunming	25.02	4.26 h
Lanzhou	36.05	4.40 h	Guangzhou	23.13	3.52 h
Zhengzhou	34.72	4.04 h	Nanjing	22.82	3.54 h
Xi’an	34.30	3.60 h	Haikou	20.03	3.75 h
Nanjing	32.00	3.94 h			

Source: Zhen [37].

**Table 2:** Environmental benefits of a solar water heater per m^2^.

Name	Emission Factor (kg/kgce)	Annual Emission Amount (kg)	Annual Environmental Benefit (¥/kg)	Total Environmental Benefits during Useful Life (¥)
1. Reduction of Harmful Gases Emission				
SO_2_	0.022	4.85	10.26	49.8
NO_2_	0.01	2.2	1.8	3.96
Smoke Dust	0.017	3.75	4.48	16.8
CO_2_ (Greenhouse Gas)	1.79	322	0.20	64.4
2. Total Environmental Benefits			75.02	750.2

Source: Huo [44].

**Table 3:** Economic benefit growth of the solar water heater industry between 2006 and 2010.

Year	Total Revenue (100 Million Yuan)	Export (100 Million US Dollars)
2006	270	1.25
2007	320	1.5
2008	465	1.8
2009	600	2.0
2010	735	2.5
Total	2390	9.05

Source: Huo [44].

**Table 4:** Employment in the solar water heater industry between 2006 and 2010.

Year	Employment (10 Thousand Persons)
2006	200
2007	250
2008	280
2009	300
2010	330

Source: Huo [44].

**Table 5:** Amount of energy saved and amount of emissions reduced by using solar water heaters between 2000 and 2010.

Year	Amount of Solar Water Heater Held (m^2^/MW_th_)	Amount of Standard Coal Saved (GWh)	Amount of Electricity Saved (GWh)	Reduced Amount of SO_2_ (10,000 Tons)	Reduced Amount of NO_2_ (10,000 Tons)	Reduced Amount of Smoke Dust (10,000 Tons)	Reduced Amount of CO_2_ (10,000 Tons)
**2000**	2600/18,200	390	108.42	12.61	5.72	9.75	837.2
**2001**	3200/22,400	480	133.44	15.52	7.04	12.0	1030.4
**2002**	4000/28,000	600	166.80	19.40	8.80	15.0	1288
**2003**	5000/35,000	750	208.50	24.25	11.0	18.75	1610
**2004**	6200/43,400	930	258.54	30.07	13.64	23.25	1996.4
**2005**	7500/52,500	1125	312.75	36.37	15.0	28.12	2415
**2006**	9000/63,000	1350	375.30	43.65	19.8	33.75	2898
**2007**	10,800/75,600	1620	450.36	52.38	23.76	40.50	3477.6
**2008**	12,500/87,500	1875	521.25	60.62	27.50	46.87	4025
**2009**	14,500/101,500	2175	604.65	70.32	31.90	54.37	4669
**2010**	16,800/117,600	2352	653.86	81.48	36.96	63.00	5045
**Total**		13,647	3195.68	446.67	201.12	345.36	29,291.60

Source: Huo [44].

**Table 6:** Present value of the total costs of using a solar water heater.

Year	Discount Factor (1 + i)^−t^ ①	Buying Price (¥) ②	Annual Maintenance Cost (¥) ③	Annual Times of Shower Taken (Times) ④	Annual Water Fee (¥) ⑤	Annual Total Costs (¥) ⑥ = ② + ③ + ⑤	Annual Present Value of Costs (¥) ⑦ = ⑥ × ①
1	0.99616	3500.00	10.00	520	192.40	3702.40	3688.20
2	0.99234		10.00	520	192.40	202.40	200.85
3	0.98854		10.00	520	192.40	202.40	200.08
4	0.98475		30.00	520	192.40	222.40	219.01
5	0.98097		30.00	520	192.40	222.40	218.17
6	0.97721		30.00	520	192.40	222.40	217.33
7	0.97346		30.00	520	192.40	222.40	216.50
8	0.96973		30.00	520	192.40	222.40	215.67
9	0.96601		30.00	520	192.40	222.40	214.84
10	0.96230		30.00	520	192.40	222.40	214.02
Present Value of Total Costs							5604.67

**Table 7:** Total annual environmental benefits of using a solar water heater.

Name	Annual Emission Amount per m^2^ Solar Water Heater Held (kg/m^2^) ①	Annual Environmental Benefit per Kilogram (¥/kg) ②	Annual Reduced Emission Amount (kg) ③ = ① × 1.96 m^2^	Annual Environmental Benefits (¥) ④ = ③ × ②
SO_2_	4.85	10.26	9.51	97.57
NO_2_	2.20	1.80	4.31	7.76
Smoke Dust	3.75	4.48	7.35	32.93
CO_2_	322.00	0.20	631.12	126.22
Total Annual Environmental Benefits				264.48

*Source*: Huo [44].

**Table 8:** Present value of the total environmental benefits of using a solar water heater.

Year	Discount Factor (1 + i)^−t^ ①	Total Annual Environmental Benefits (¥) ②	Present Value of Total Annual Environment Benefits (¥) ③ = ① × ②
1	0.99616	264.48	263.47
2	0.99234	264.48	262.46
3	0.98854	264.48	261.45
4	0.98475	264.48	260.45
5	0.98097	264.48	259.45
6	0.97721	264.48	258.45
7	0.97346	264.48	257.46
8	0.96973	264.48	256.47
9	0.96601	264.48	255.49
10	0.96230	264.48	254.51
Present Value of Total Environmental Benefits			2589.67

**Table 9:** Present value of the total costs of using an electronic water heater.

Year	Discount Factor (1 + i)^−t^ ①	Buying Price (¥) ②	Annual Maintenance Cost (¥) ③	Annual Times of Shower Taken (Times) ④	Annual Water Fee (¥) ⑤	Annual Electricity Fee (¥) ⑥	Annual Total Costs (¥) ⑦ = ② + ③ + ⑤ + ⑥	Annual Present Value of Costs (¥) ⑧ = ⑦ × ①
**1**	0.99616	1756.00	40.00	520	192.40	182.00	2170.40	2162.08
**2**	0.99234		40.00	520	192.40	182.00	414.40	411.23
**3**	0.98854		40.00	520	192.40	182.00	414.40	409.65
**4**	0.98475		40.00	520	192.40	182.00	414.40	408.08
**5**	0.98097		40.00	520	192.40	182.00	414.40	406.51
**6**	0.97721		40.00	520	192.40	182.00	414.40	404.96
**7**	0.97346		40.00	520	192.40	182.00	414.40	403.40
**8**	0.96973		40.00	520	192.40	182.00	414.40	401.85
**9**	0.96601		40.00	520	192.40	182.00	414.40	400.31
**10**	0.96230		40.00	520	192.40	182.00	414.40	398.78
**Total**	9.79147	Present Value of Electronic Fees		9.79147 × 182.00 = 1782.05				
Present Value of Total Costs								5806.85

**Table 10:** Present value of the total costs of using a coal gas water heater.

Year	Discount Factor (1 + i)^−t^ ①	Buying Price (¥) ②	Annual Maintenance Cost (¥)③	Annual Times of Shower Taken (Times) ④	Annual Water Fee (¥) ⑤	Annual Coal Gas Fee (¥) ⑥	Annual Total Costs (¥) ⑦ = ② + ③ + ⑤ + ⑥	Annual Present Value of Costs (¥) ⑧ = ⑦ × ①
**1**	0.99616	2000.00	40.00	520	192.40	205.40	2437.80	2428.45
**2**	0.99234		40.00	520	192.40	205.40	437.80	434.45
**3**	0.98854		40.00	520	192.40	205.40	437.80	432.78
**4**	0.98475		40.00	520	192.40	205.40	437.80	431.12
**5**	0.98097		40.00	520	192.40	205.40	437.80	429.47
**6**	0.97721		40.00	520	192.40	205.40	437.80	427.82
**7**	0.97346		40.00	520	192.40	205.40	437.80	426.18
**8**	0.96973		40.00	520	192.40	205.40	437.80	424.55
**9**	0.96601		40.00	520	192.40	205.40	437.80	422.92
**10**	0.96230		40.00	520	192.40	205.40	437.80	421.30
Total	9.79147	Present Value of Coal Gages Fee			9.79147 × 205.40 = 2011.17			
Present Value of Total Costs								6279.04
